# Printability and microstructure of directed energy deposited SS316l-IN718 multi-material: numerical modeling and experimental analysis

**DOI:** 10.1038/s41598-022-21077-8

**Published:** 2022-10-05

**Authors:** Reza Ghanavati, Homam Naffakh-Moosavy, Mahmoud Moradi, Mohsen Eshraghi

**Affiliations:** 1 Department of Materials Engineering, Tarbiat Modares University (TMU), PO Box 14115-143, Tehran, Iran; 2 Faculty of Arts, Science and Technology, University of Northampton, Northampton, NN1 5PH UK; 3 Department of Mechanical Engineering, California State University, Los Angeles, CA 90032 USA

**Keywords:** Mechanical engineering, Composites, Metals and alloys

## Abstract

In the present paper, the interrelated aspects of additive manufacturing-microstructure-property in directed energy deposition of SS316L-IN718 multi-material were studied through numerical modeling and experimental evaluation. The printability concept and solidification principles were used for this purpose. The printability analysis showed that the SS316L section is more susceptible to composition change and lack of fusion, respectively due to the high equilibrium vapor pressure of manganese and the more efficient heat loss in the initial layers. However, the IN718 section is more prone to distortion due to the formation of a larger melt pool, with a maximum thermal strain of 3.95 × 10^−3^ in the last layer. As the process continues, due to heat accumulation and extension of the melt pool, the cooling rate decreases and the undercooling level increases, which respectively result in coarser microstructure and more instability of solidification front in the build direction, as also observed in the experimental results. The difference is that the dendritic microstructure of the IN718 section, due to the eutectic reaction L → γ + Laves, is formed on a smaller scale compared to the cellular microstructure of the SS316L section. Also, the decrease in cooling rate caused the secondary phase fraction in each section (delta ferrite in SS316L and Laves in IN718) to increase almost linearly. However, the hardness calculation and measurement showed similarly, even though with the transition from SS316L to IN718 the hardness is significantly increased due to higher yield strength of the matrix and the presence of Laves intermetallic phase (~ 260 HV0.3), the hardness in each section decreases slightly due to the coarsening of the microstructure from the initial layer to the final.

## Introduction

Nowadays, many engineering structures are made up of multiple materials. This is because meeting various service and performance requirements can hardly be satisfied by one material. Therefore, it is often necessary to use dissimilar materials together. This has led to the generalization of the concept of “multi-material structures” in the engineering sciences. Hence, the role of multi-material structures has been proven and many studies have been done on them in the past. Nevertheless, the evolution of modern additive manufacturing (AM) technology, with distinct advantages such as the ability to produce integrated near net shape complex parts in one step, cost-effectiveness for small-scale production, and high-level customization, has eliminated many of the limitations of conventional manufacturing methods and opened up new dimensions to the development and research of multi-materials^1,2^. From the subset of metal additive manufacturing processes as the fastest growing sector of AM today^3^, the directed energy deposition (DED) and the powder bed fusion (PBF) are both of interest in the fabrication of multi-materials. However, DED has become more popular due to its greater flexibility in changing chemical composition during processing^4^. According to the studies conducted so far, metallic multi-materials processed by DED can be classified based on the type of alloy (mainly Ti, Fe, and Ni alloys) and build strategy (bimetallic, functionally graded, and hybrid materials)^5^.

Stainless steels/nickel-based superalloys multi-materials are of the most widely used combinations in critical energy industries due to their cost-performance balance tailored to service requirements^6^. Therefore, given this issue and the inherent characteristics of AM, some of which were mentioned above, various research studies have been done on the additive manufacturing of this type of multi-material in recent years. Lin et al.^7,8^ studied the microstructure evolution and phase formation in laser rapid forming (LRF) of SS316L/Rene88DT graded material. Shah et al.^9^ investigated the effect of laser direct metal deposition (LDMD) parameters on the development of SS316L/IN718 graded structure. Savitha et al.^10^ in a study on additive manufacturing of SS316/IN625 dual materials observed that the yield strength is always comparable to the weaker component (SS316), while Zhang et al.^11^ in a similar study obtained the yield strength and tensile strength of gradient samples close to IN625 and SS316L, respectively. Carroll et al.^12^ in determining the cause of cracking in a graded structure fabricated from SS304L and IN625 by DED, demonstrated the role of metal monocarbides in the form of (Mo, Nb)C using thermodynamic modeling by CALculation of PHAse Diagrams (CALPHAD) method. Su et al.^13^ investigated the effect of various gradient composition in laser additive manufacturing of SS316L/IN718 functionally graded material. They reported that the best combination of mechanical properties (tensile strength of 527.05 MPa and elongation of 26.21%) were obtained with a composition change step of 10%. In another study, Kim et al.^14^ observed that the formation of defects (pores and cracks) occurs in certain chemical composition ranges of the SS316L/IN718 structure affected by ceramic oxides and their subsequent propagation in the direction of intermetallic and carbide compounds. Moreover, thermal and residual stresses concentrated at the grain boundaries exacerbated the formation of these defects.

An overview of previous studies suggests that most of the efforts have been focused on the experimental study of one aspect of the processing, structure, and properties (materials paradigm) of stainless steel/nickel-based superalloys multi-material. However, given the importance of all the interrelated aspects of the materials paradigm, a comprehensive understanding and the possibility of predicting them in the additive manufacturing of multi-materials can lead to a better framework for controlling them. In addition, the numerical approach can provide a smoother path to achieve that by reducing the time and cost spent on various experiments. Therefore, in the present study, the concept of printability was used through finite element modeling to investigate processing challenges of 316 low carbon stainless steel (SS316L)-Inconel 718 (IN718) multi-material by DED. Also, solidification principles were applied by the modeling results to evaluate the microstructure characteristics and estimate the properties of the multi-material. Besides, experimental studies have been used to better support and analyze numerical results.

## Numerical modeling

**Figure 1:**
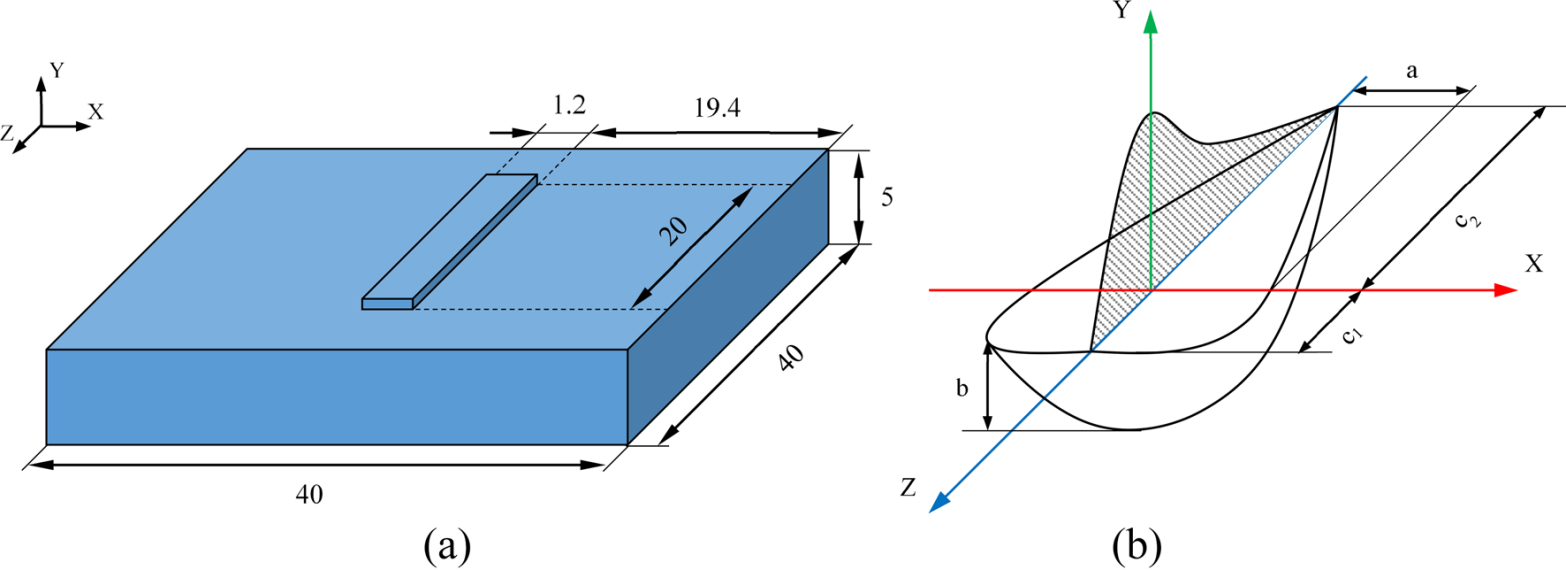
(**a**) The geometry of the single-layer deposition (mm). The deposition thickness was considered 0.8 mm according to the results obtained in experimental evaluations. (**b**) The double ellipsoidal heat source model and its parameters.

In the numerical study, heat transfer modeling during the manufacturing process of multi-material structure, consisting of 7 layers of SS316L alloy and 7 layers of IN718 alloy, was performed by the finite element method (FEM). First, the geometry of a single layer deposition (Fig. 1a) was modeled and after finding the appropriate mesh size, the modeling procedure was repeated using the element birth and death technique for the subsequent layers until the design was completed. It is worth noting that the surfaces of the deposited layers were assumed flat for simplification. The governing equation of the problem (transient heat transfer) can be expressed as follows:

1 $$\frac{\partial }{\partial x}\left(k\frac{\partial T}{\partial x}\right)+\frac{\partial }{\partial y}\left(k\frac{\partial T}{\partial y}\right)+\frac{\partial }{\partial z}\left(k\frac{\partial T}{\partial z}\right)+\dot{Q}=\rho {C}_{p}\frac{\partial T}{\partial t}$$

where $x$, $y$, and $z$ are the transverse, building, and laser scanning directions, respectively, and *k* is the thermal conductivity, $T$ is the temperature, $\rho$ is the density, ${C}_{p}$ is the specific heat, $t$ is the time, and $\dot{Q}$ is the rate of internal heat generation (here by phase change). Also, the matrix form of the differential Eq. (1) can be written as follows:

2 $$\rho {C}_{p}\frac{\partial T}{\partial t}={\left[L\right]}^{T}\left(\left[D\right]\left[L\right]T\right)+\dot{Q}$$

where, $\left[L\right]$ and $\left[D\right]$ are the vector differential operator and conduction coefficient matrices, respectively, which are expressed as:

3 $$\left[L\right]=\left[\begin{array}{ccc}\frac{\delta }{\delta x}& 0& 0\\ 0& \frac{\delta }{\delta y}& 0\\ 0& 0& \frac{\delta }{\delta z}\end{array}\right]$$

4 $$\left[D\right]=\left[\begin{array}{ccc}{k}_{xx}& 0& 0\\ 0& {k}_{yy}& 0\\ 0& 0& {k}_{zz}\end{array}\right]$$

The initial and boundary conditions are expressed in Eqs. (5) and (6), respectively:

5 $$T\left(x,y,z,0\right)={T}_{a}$$

6 $$k\frac{\partial T}{\partial n}+{h}_{c}\left(T-{T}_{a}\right)+\sigma \varepsilon \left({T}^{4}-{T}_{a}^{4}\right)-q=0$$

where ${T}_{a}$ is the ambient temperature (298 K), $n$ is surface normal, ${h}_{c}$ is convection coefficient, $\sigma$ is the Stephen–Boltzmann constant, $\varepsilon$ is emissivity and $q$ is the heat flux generated by the laser beam. Obviously, for surfaces other than those irradiated by the laser beam, the amount of heat flux ($q$) is zero in Eq. (6) and also the heat loss due to radiation (third term) can be ignored. It should be noted that for convenience and to avoid non-linearization due to the radiation heat loss, the third term in Eq. (6) was removed, and instead of ${h}_{c}$ a previously developed effective heat transfer coefficient ($h$)^15^ was used, which is a combination of both:

7 $$h=2.4\times {10}^{-3}\varepsilon {T}^{1.61}$$

where the units of $h$, and $T$ are W/m^2^ K and K, respectively.

Also, to model the laser heat source, owing to the necessity of using the laser conduction mode in the AM process and the experimental observation of the melt pool geometry in the cross-section, a double ellipsoidal power density distribution was considered as shown in Fig. 1b^16^. In this model, the power density distribution in the front and rear quadrants are defined by the following equations, respectively:

8 $$q\left(x,y,z,t\right)=\frac{6\sqrt{3}{f}_{f}Q}{abc\pi \sqrt{\pi }}\mathit{exp}\left(-3{x}^{2}/{a}^{2}\right)\mathit{exp}\left(-3{y}^{2}/{b}^{2}\right)exp\left(-3{\left[z+vt\right]}^{2}/{{c}_{1}}^{2}\right)$$

9 $$q\left(x,y,z,t\right)=\frac{6\sqrt{3}{f}_{r}Q}{abc\pi \sqrt{\pi }}\mathit{exp}\left(-3{x}^{2}/{a}^{2}\right)\mathit{exp}\left(-3{y}^{2}/{b}^{2}\right)\mathit{exp}\left(-3{\left[z+vt\right]}^{2}/{{c}_{2}}^{2}\right)$$

where $Q$ is the effective laser power (W), $v$ is the scanning velocity (m/s), and $a$, $b$, ${c}_{1}$, and ${c}_{2}$ are independent values in determining how the heat flux is distributed. ${f}_{f}$ and ${f}_{r}$ are the heat fractions for the front and rear quadrants, respectively, with a relation of ${f}_{f}$ + ${f}_{r}$ = 2 between them.

**Figure 2:**
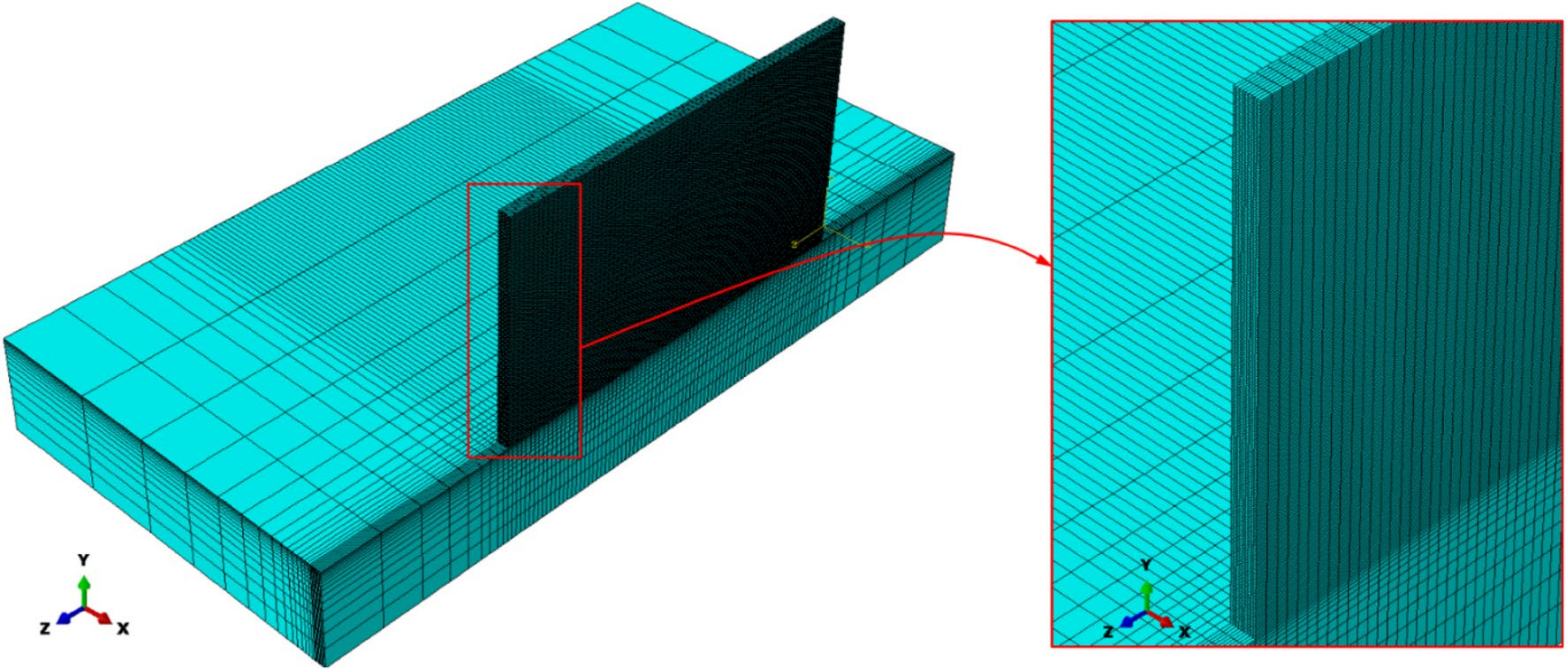
The mesh system used for the final model (14-layer structure).

In this study, the FEM software ABAQUS v. 6.14 was employed to solve the governing heat transfer equation. To increase the solution accuracy, the thermophysical properties as a function of temperature for SS316L and IN718 alloys were extracted from Refs.^17,18^, respectively, and defined in the software. Also, to take into account the heat transfer due to fluid flow in the melt pool, it was assumed that the thermal conductivity of materials above the solidus temperature up to 3000 K increases linearly with a factor of about three^19^. Figure 2 shows the mesh system consisting of DC3D8 hexahedral elements used for the final model after the mesh sensitivity analysis. It should be noted that due to the geometric symmetry, only half of it (longitudinal section) was modeled to reduce the calculations. Also, since there is a high thermal gradient in the deposition path, finer meshes were used in that area, as shown in Fig. 2. Finally, 191,808 elements and 226,820 nodes were used for the modeling. Besides, The ABAQUS user subroutine DFLUX was utilized to apply the laser heat flux according to the double ellipsoidal distribution model (Eqs. 8, 9) as a function of the location and time.

## Experimental evaluation

SS316L and IN718 gas-atomized powders with average diameters of 110 and 70 µm, respectively, and SS316L substrate with dimensions of 40 × 40 × 5 mm were used as raw materials. The chemical compositions of the powders are presented in Table 1. A DED additive manufacturing machine with specifications of 1 kW continuous-wave fiber laser of 1080 nm wavelength and ~ 1 mm spot diameter (YFL-1000 model, National Laser Center of Iran), four-channel nozzle delivering powder coaxial with the laser beam, twin powder feeder (model PF 200, Noura, Iran), carrier and shielding Ar gas, and CNC table with 3 degrees of freedom, was employed to fabricate the multi-material sample.

**Table 1:** Chemical compositions of SS316L and IN718 powders (wt%).

Materials	Fe	Ni	Cr	Mn	Mo	Nb	Al	Ti
SS316L	Base	12.45	17.48	1.75	2.47	–	–	–
IN718	19.32	Base	19.09	–	3.21	5.37	1.83	1.21

**Figure 3:**
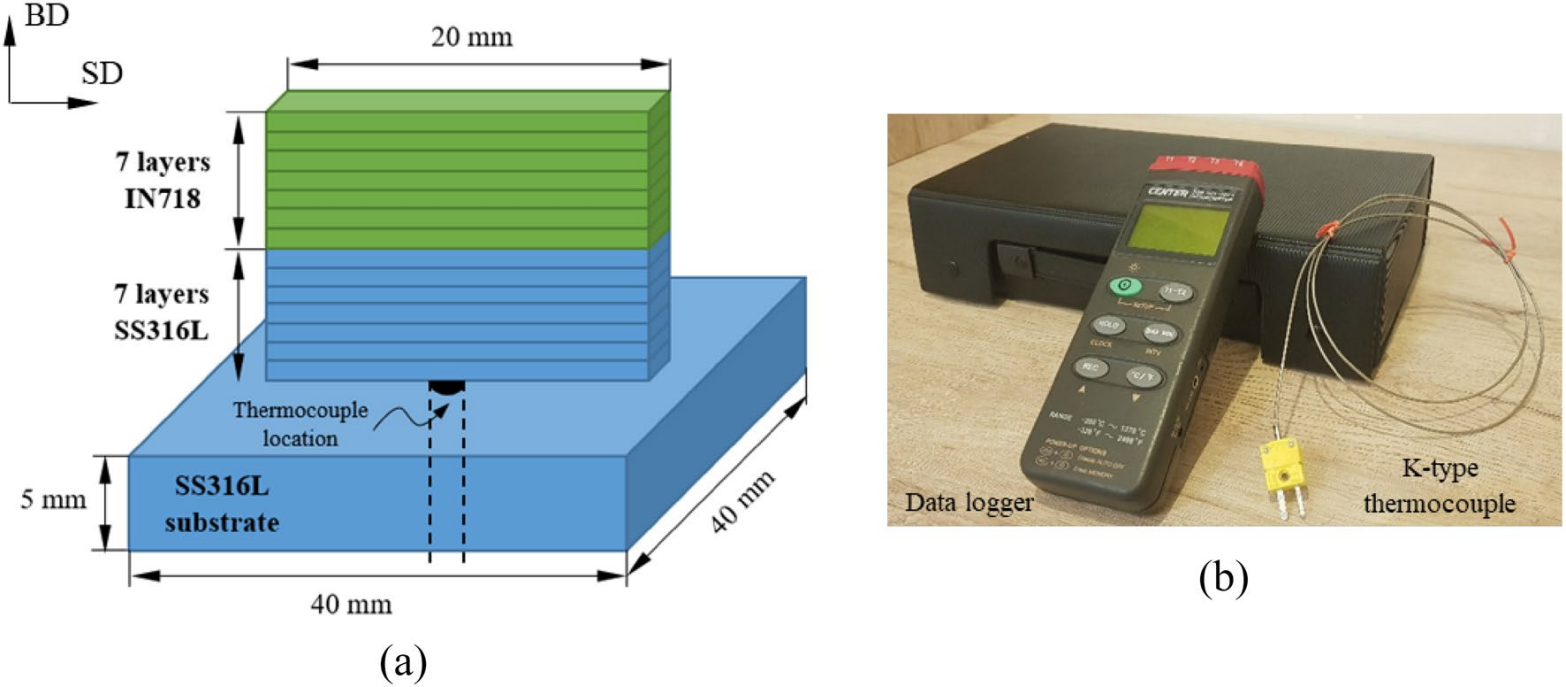
(**a**) Schematic of the multi-material structure (the thermocouple location: in the middle of the laser scanning/deposition path and 1 mm below the substrate surface). (**b**) Thermocouple and data logger used to measure temperature during the process.

**Figure 4:**
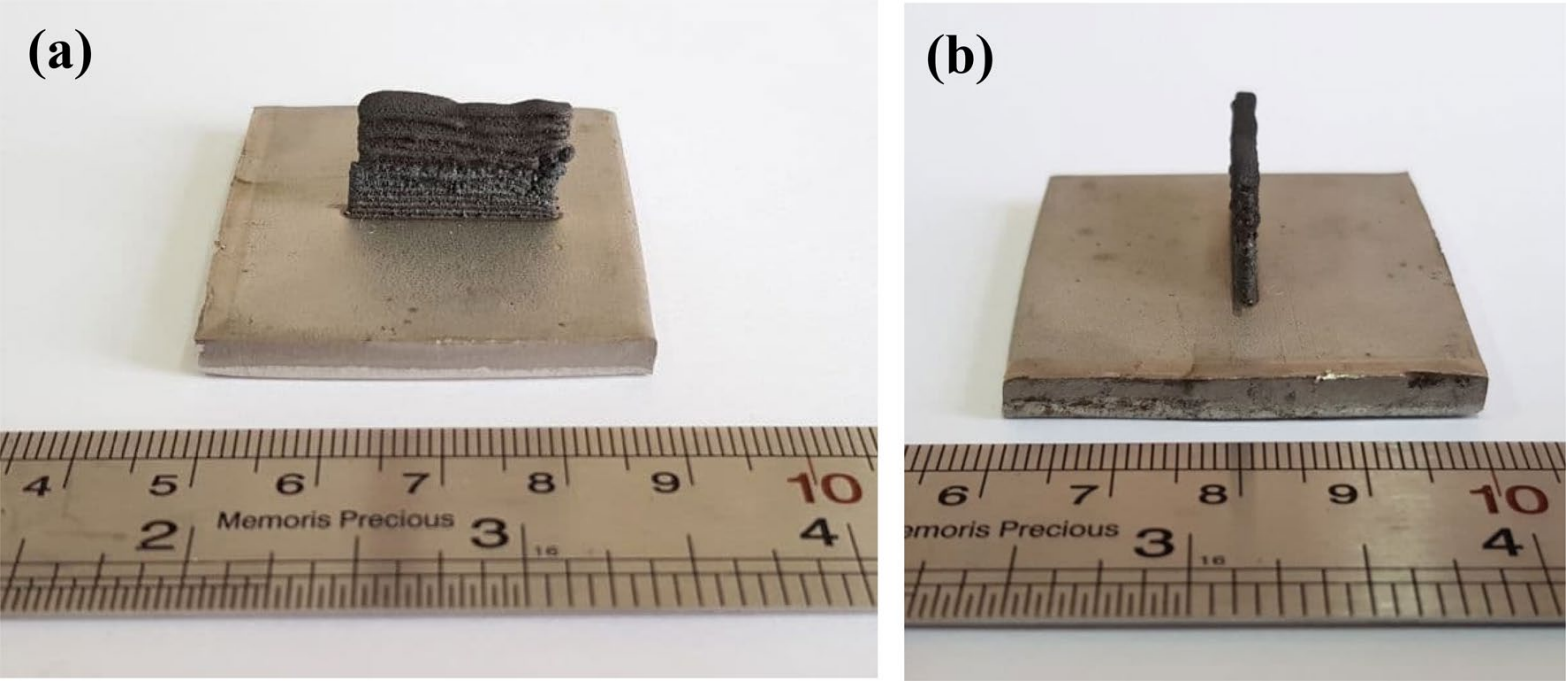
(**a**) Front view and (**b**) side view of the SS316L-IN718 multi-material structure fabricated according to Fig. 3a.

The multi-material structure was fabricated according to Fig. 3a as a unidirectional thin-wall consisting of 7 layers of SS316L and 7 layers of IN718 under the processing parameters presented in Table 2. To validate the FE model, the thermal history (temperature–time diagram) was recorded during the process using a K-type thermocouple (Fig. 3b) embedded in the middle and below the deposition path. The results were compared with the thermal history obtained from the corresponding location in the simulated model. Figure 4 shows the SS316L-IN718 multi-material structure fabricated according to the design shown in Fig. 3a.

**Table 2:** DED processing parameters.

Parameter	Value
Laser power (W)	250
Scanning velocity (mm min^−1^)	300
Powder feed rate (g min^−1^)	27.5
Axial gas flow (L min^−1^)	3
Carrier gas flow (L min^−1^)	1.5
Standoff distance (mm)	15
Z-step (mm)	0.8

To study the metallurgical features of the multi-material, a cross-section was cut from the mid-length of the structure using an electric discharge machine (EDM) and after preparing its surface by standard metallographic methods, it was etched by holding in 15 mL HCl + 5 mL HNO_3_ solution for 10 s. The optical microscopy (Olympus, Japan) and scanning electron microscopy (FEI ESEM QUANTA 200, USA) were used to qualitatively evaluate the microstructure and quantify its characteristics by ImageJ software. Also, a semi-quantitative evaluation of the constituent elements distribution and the composition of possible phases in the microstructure was performed by an X-ray dispersive energy spectroscope (EDAX EDS Silicon Drift 2017, USA) used in the SEM. The hardness variations in the build direction were measured using a Vickers microhardness tester (Buehler, Japan) on the prepared section at intervals of every 500 µm with a 300 gf load and a dwell time of 10 s. As well, three microhardness measurements were taken at each height of the structure and the mean value was reported to minimize the measurement error.

## Results and discussion

### Printability analysis

The concept of printability is the ability of an alloy to resist distortion, chemical composition changes, and lack of fusion as common defects in the additive manufacturing of metal parts^20^. Similar to the universal concept of weldability in the science of welding metallurgy^21^, the development of printability can facilitate the challenging selection of the printing process and its parameters for the desired alloy(s) by establishing a comprehensive database and reducing the risk of common defects without additional cost and time^22^. In this section, after validating the FE model and presenting the initial results, the distortion, chemical composition changes, and lack of fusion defects will be examined for the multi-material.

**Figure 5:**
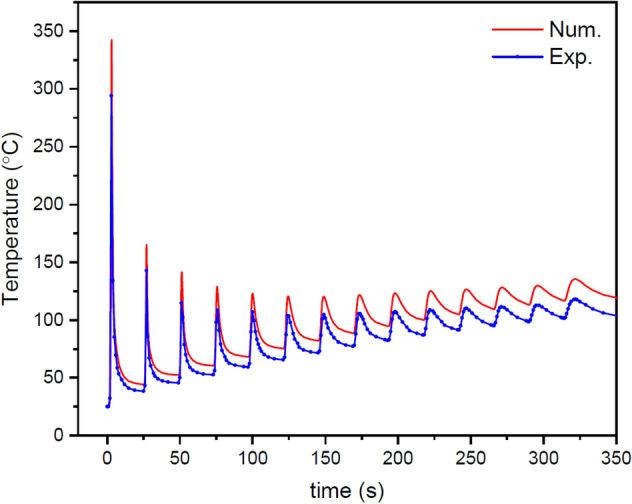
Comparison of thermal histories obtained from the FE model (red) and experimental measurements (blue) at the same location.

**Figure 6:**
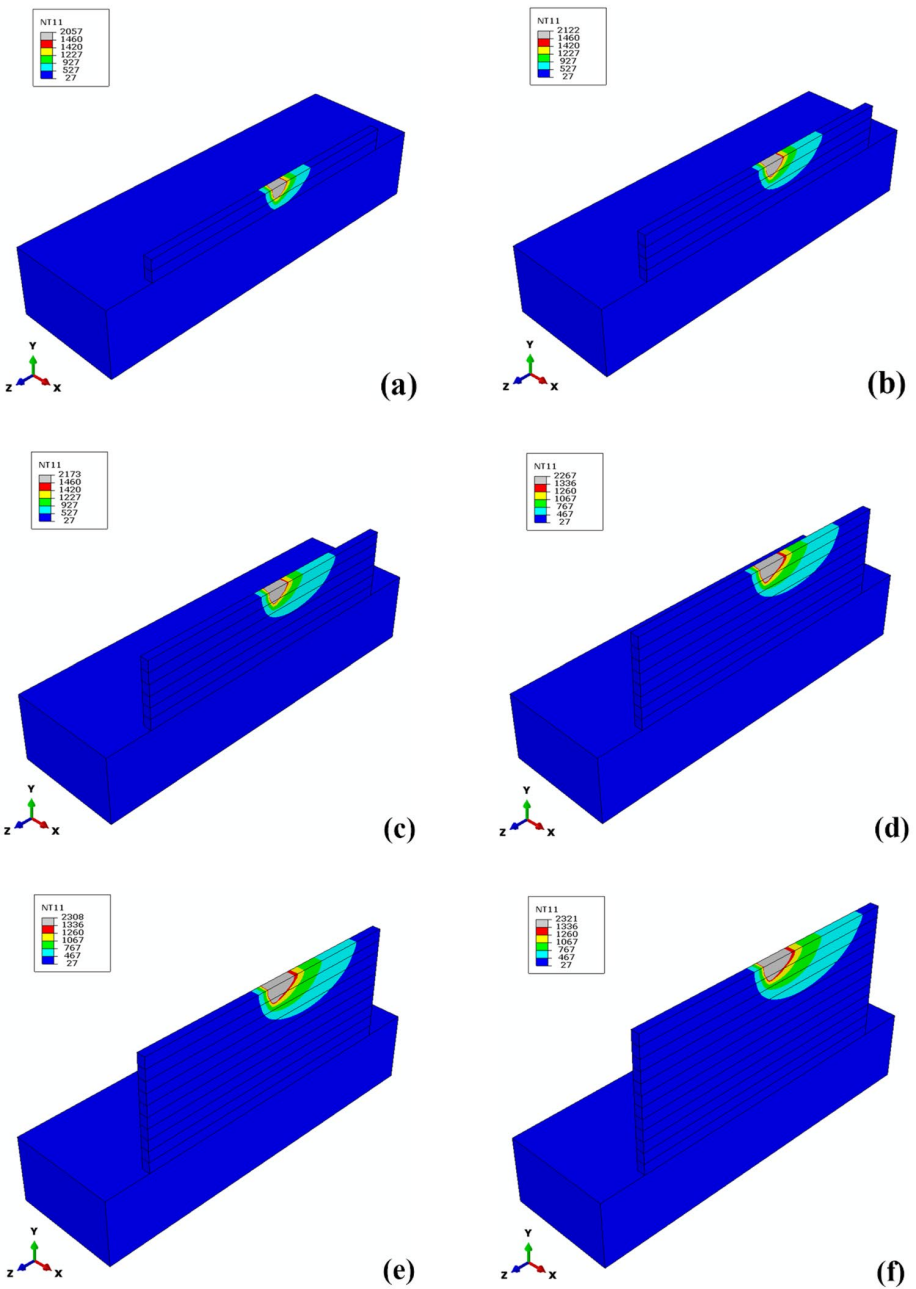
Temperature field (℃) and the simulated melt pool (gray zone) in the mid-length of layers (**a**) 2, (**b**) 4, (**c**) 6, (**d**) 8, (**e**) 10, and (**f**) 12.

Figure 5 shows a comparison of the thermal histories obtained from the thermal model and measured by the thermocouple in the same location (in the middle of the deposition length and 1 mm below the substrate surface). As can be seen, the acceptable difference between the numerical and experimental results indicates the proper accuracy of the thermal model, and therefore other results that can be derived from it can be trusted. Figure 6 shows the longitudinal section of simulated melt pools for even layers in the SS316L-IN718 multi-material structure. What can be understood is that as the process progresses, the melt pool size, the extent of re-melting of the previously deposited layers, and the peak temperature increase, which is to be expected given the physics of the problem. The mentioned phenomena can be attributed to two factors: (a) reducing the heat sink effect by the substrate as the process progresses (heat accumulation) and (b) the difference in thermal properties of the base alloys, such as a certain difference in their solidification temperature ranges (SS316L: 1460–1420 ℃ and IN718: 1336–1260 ℃). What is important is the influence of these factors on the printability indicators and the microstructural aspects of the multi-material, which are discussed in this section and the next section, respectively.

**Figure 7:**
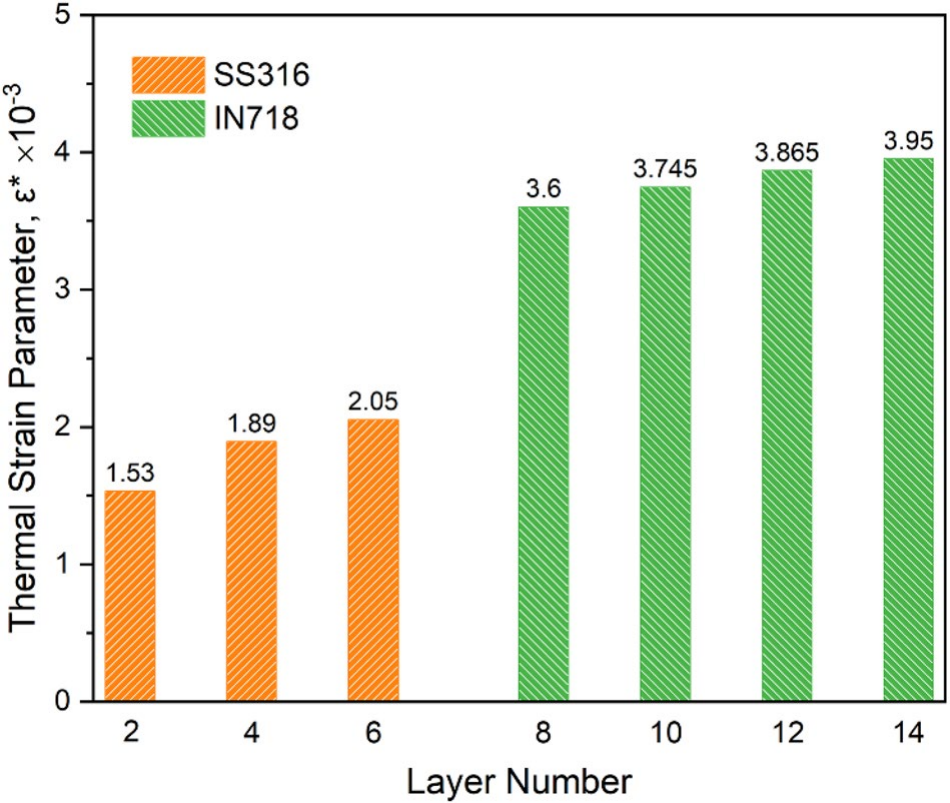
Thermal strain parameter (${\varepsilon }^{*}$) variations in different layers of multi-material structure.

Thermal distortion during the process depends on the alloy properties and the process parameters. The tendency to distortion can be calculated using the maximum thermal strain criterion. Recently, a non-dimensional thermal strain parameter, ${\varepsilon }^{*}$ (representative of the maximum thermal strain), as a function of alloy properties and process parameters has been developed based on Buckingham's π-theorem^20^:

10 $${\varepsilon }^{*}=\frac{\beta \Delta T}{EI}\frac{t}{F\sqrt{\rho }}{H}^{3/2}$$

where $\beta$ is the volume expansion coefficient, $\Delta T$ is the difference between peak temperature and ambient temperature, $t$ is the deposition time, $H$ is the heat input per unit length, $EI$ is the flexural rigidity, and $\rho$ is the alloy density. The Fourier number $F$, which represents the ratio of heat transfer to heat accumulation, can also be rewritten as $\alpha /vw$, where $\alpha$ is the thermal diffusion coefficient, $v$ is the beam scanning velocity, and $w$ is the length of the melt pool. As can be seen in Fig. 7, with increasing number of layers, the thermal strain generally increases due to the weakening of heat transfer from the melt pool to the substrate and consequently the higher temperature difference ($\Delta T$). More importantly, by changing the material from SS316L to IN718, the increase in thermal strain is more noticeable and accompanied by a mutation, the reason for which can be traced to the difference in properties of the two alloys. As mentioned earlier, the IN718 alloy with a lower solidification temperature range results in a larger melt pool (compare Fig. 6d–f to (a–c)). In other words, with a completely different increase in melt pool length ($w$), the much smaller Fourier number ($F$) is placed in Eq. (10). Therefore, a higher thermal strain is accumulated the IN718 section of the multi-material structure. This indicates that the IN718 section is more sensitive to thermal strain and should be given priority in adjusting the AM process parameters to reduce thermal strain based on Eq. (10).

**Figure 8:**
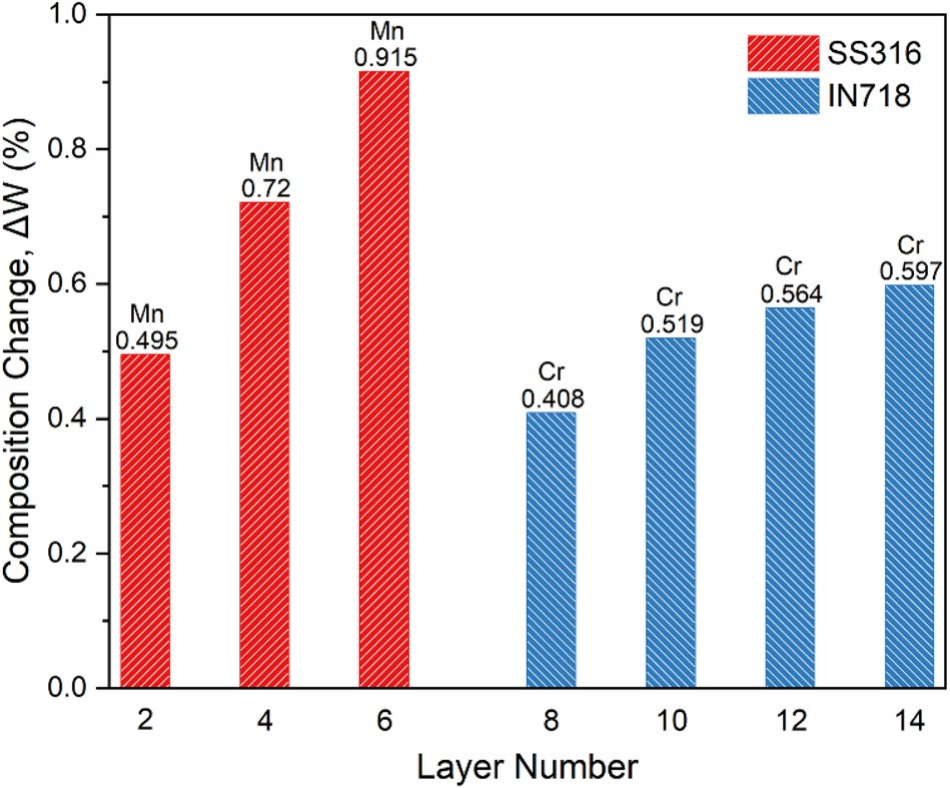
Composition change due to vaporization for elements with the highest vapor pressure in different layers of the multi-material structure.

Since some alloying elements have higher vapor pressure than others, selective vaporization of alloying elements in AM is highly probable which can lead to a considerable change in the chemical composition of the alloy and thus a decrease in its properties such as strength, hardness, and corrosion resistance. The Langmuir equation can be used to estimate the vaporization fluxes of alloying elements, ${J}_{i}$^20^:

11 $${J}_{i}=\frac{\lambda {P}_{i}}{\sqrt{2\pi {M}_{i}RT}}$$

where ${P}_{i}$ is the vapor pressure of element $i$ over the alloy, ${M}_{i}$ is the molecular weight of element $i$, $R$ is the gas constant, $T$ is the temperature, and $\lambda$ (= 0.05) is a positive fraction related to the condensation of vaporized atoms. Also, the following equation can be used to calculate the amount of vaporized material, $\Delta {m}_{i}$:

12 $$\Delta {m}_{i}=\frac{L{A}_{s}{J}_{i}}{v}$$

where $v$ is the beam scanning velocity, $L$ is the deposition length, and ${A}_{s}$ is the surface area of the melt pool. Given the volume of deposited material ($V$), the weight percentage of element $i$ after vaporization (${W}_{f}$) can be obtained by Eq. (13):

13 $${W}_{f}=\frac{V\rho {W}_{i}-\Delta {m}_{i}}{V\rho -\sum \Delta {m}_{i}}$$

where $\rho$ is the density and ${W}_{i}$ is the initial weight percentage of element $i$ in the powder. Figure 8 shows the composition change of the most volatile alloying elements (Mn in SS316L and Cr in IN718) in different layers of the multi-material structure due to vaporization during the DED process. In each section, as the number of layers increases, the loss of alloying elements by vaporization increases due to the higher peak temperature. However, despite higher temperatures experienced in the upper section of the multi-material (i.e. IN718), the composition change in the SS316L section is more severe for the Mn element due to its higher equilibrium vapor pressure. It can be concluded that in the multi-material structure, the SS316L section is more susceptible to composition change, and to minimize it, it should be given priority in controlling the process parameters according to Eq. (13).

**Figure 9:**
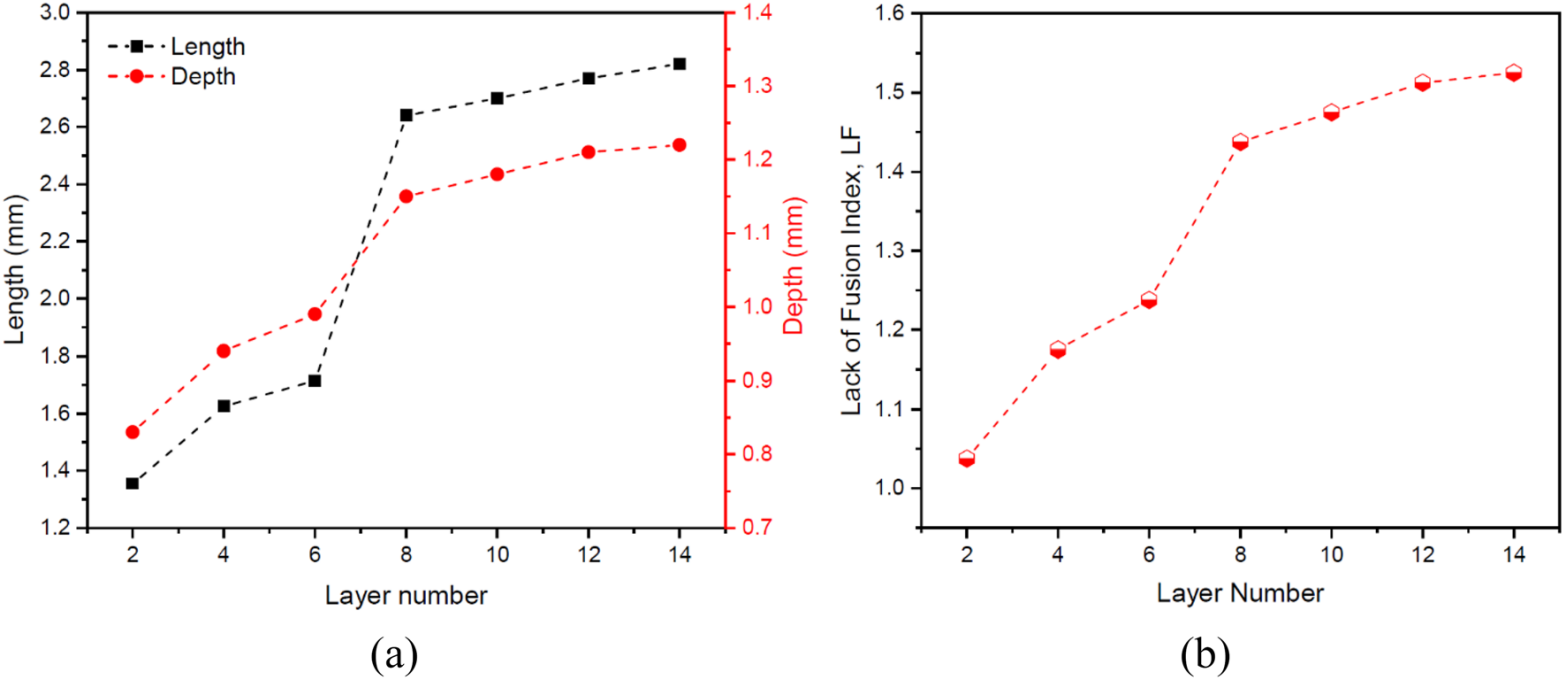
The variations of (**a**) the length and depth of the melt pool and (**b**) the $LF$ index in the build direction.

Although the penetration depth is affected by the processing conditions, different alloys in the same processing conditions show different penetration depths due to their unique thermophysical properties, which indicates different susceptibility of each to lack of fusion defect. The sufficient fusion and proper interlayer bonding can be measured by the simple index of lack of fusion, $LF$^20^:

14 $$LF=\frac{d}{h}$$

where $d$ is the penetration depth of the melt pool, and $h$ is the thickness of the deposited layer. To get a proper bonding between the layers, the penetration depth must be always greater or equal to the layer thickness or in other words *LF* must be greater or equal to 1. Increasing the dimensions of the melt pool in the upper layers of the multi-material, which can be seen in Fig. 9a by the two indicators of length and depth of the melt pool, reduces the probability of lack of fusion. Therefore, as Fig. 9b shows, the lack of fusion index ($LF$) has an increasing trend with increasing the number of layers due to heat accumulation, and more specifically, in the transition from SS316L to IN718, this trend is distinguished by a greater slope due to the lower solidification temperature range of IN718. In other words, in the SS316L section and especially in the initial layers, lack of fusion defect is more likely to occur, resulting in inadequate bonding between the layers. Therefore, this section of the multi-material structure plays a decisive role in adjusting the process parameters according to Eq. (14) to achieve an integrated structure without lack of fusion.

### Microstructure analysis

**Figure 10:**
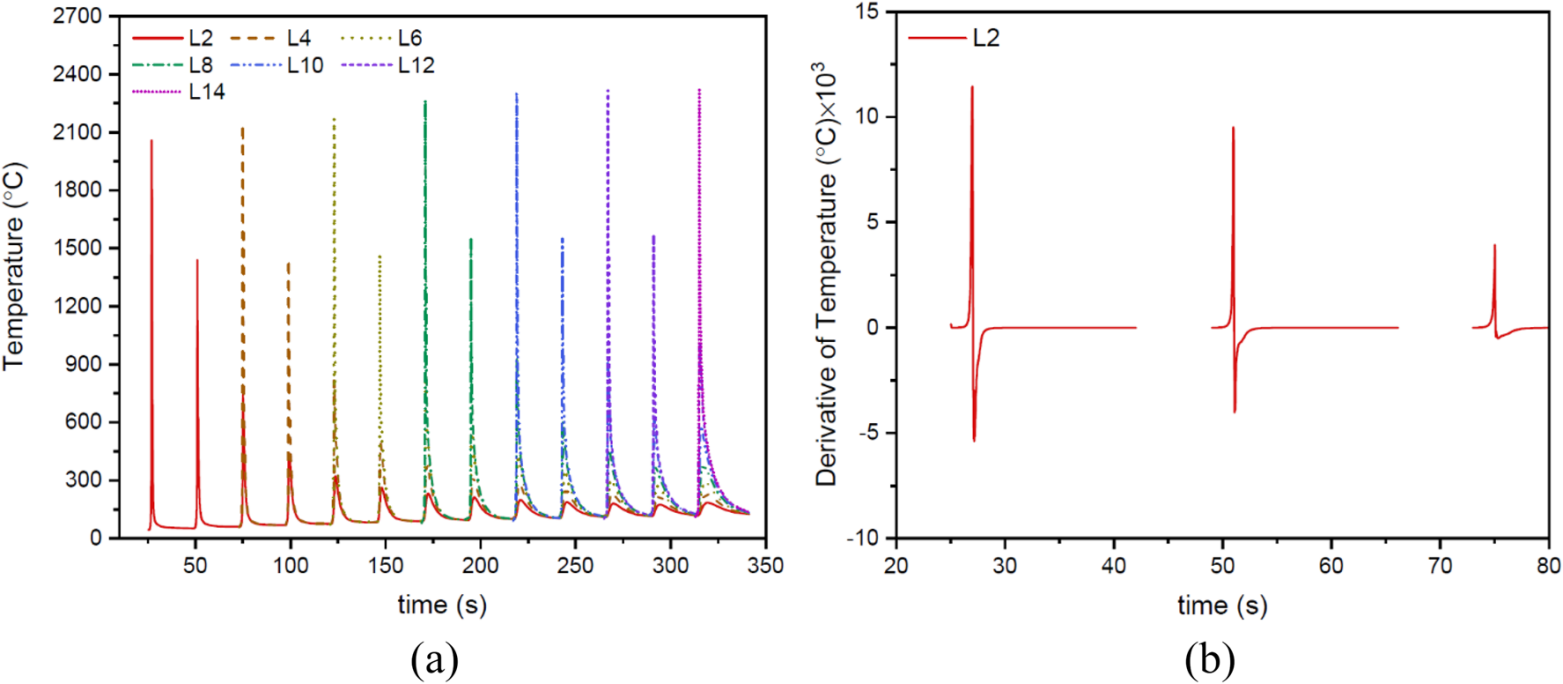
(**a**) Thermal cycles in the middle position of the lengths of layers 2, 4, 6, 8, 10, 12, and 14. (**b**) The first-order derivative of temperature with respect to time for the thermal cycle of layer 2 in (**a**).

Figure 10a shows the thermal cycles in the mid-length of layers 2, 4, 6, 8, 10, 12, and 14. The thermal cycles are unique to each layer (such in terms of peak temperature and reheating times), which can play an important role in determining the features of the layers, especially in the micro scale. To compare and discuss better, the time derivative of the temperature for each thermal cycle (as in Fig. 10b for the thermal cycle of layer 2) was used to obtain quantitative indicators, such as cooling rate.

**Figure 11:**
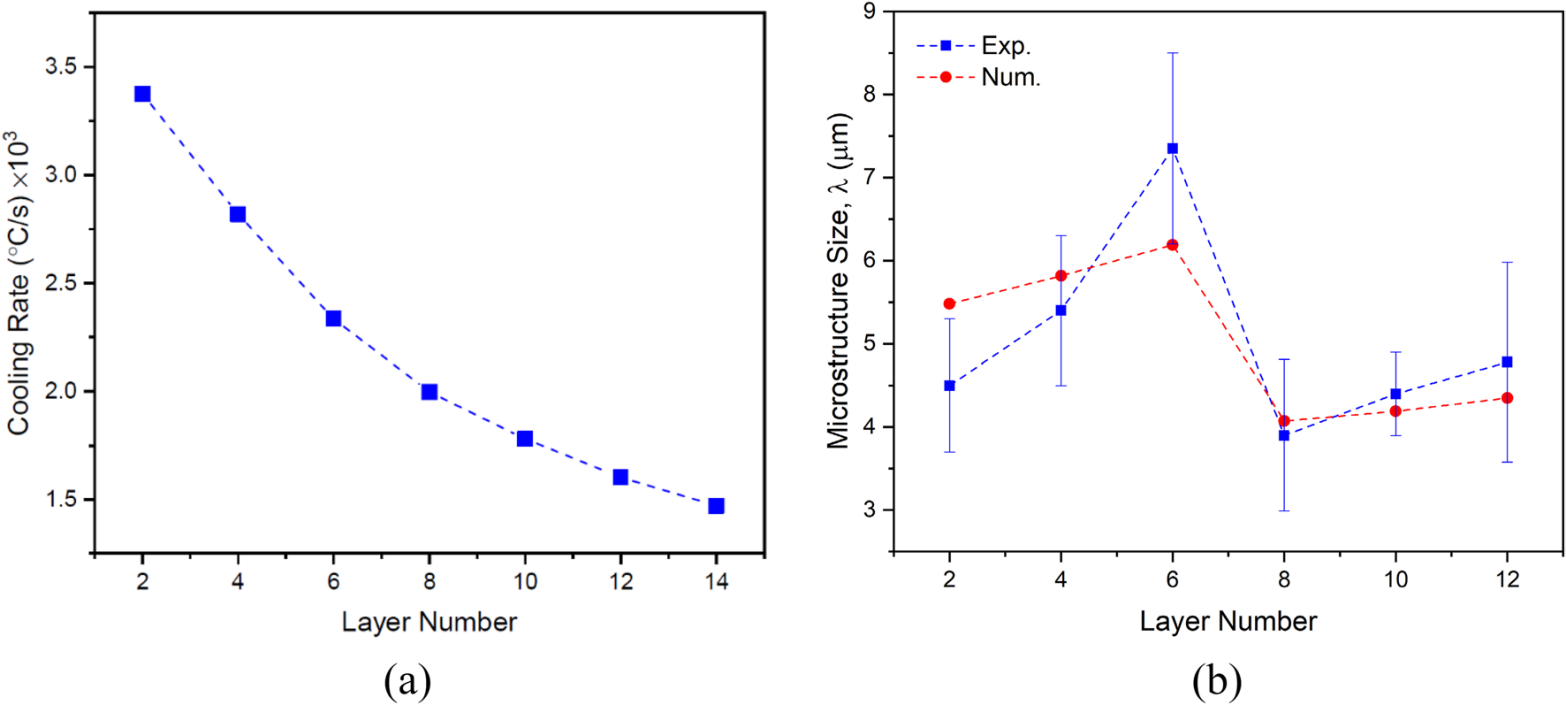
The variations of (**a**) the cooling rate in the solidification range and (**b**) the microstructure size (i.e. cellular/dendritic arm spacing, $\lambda$) in the build direction.

**Figure 12:**
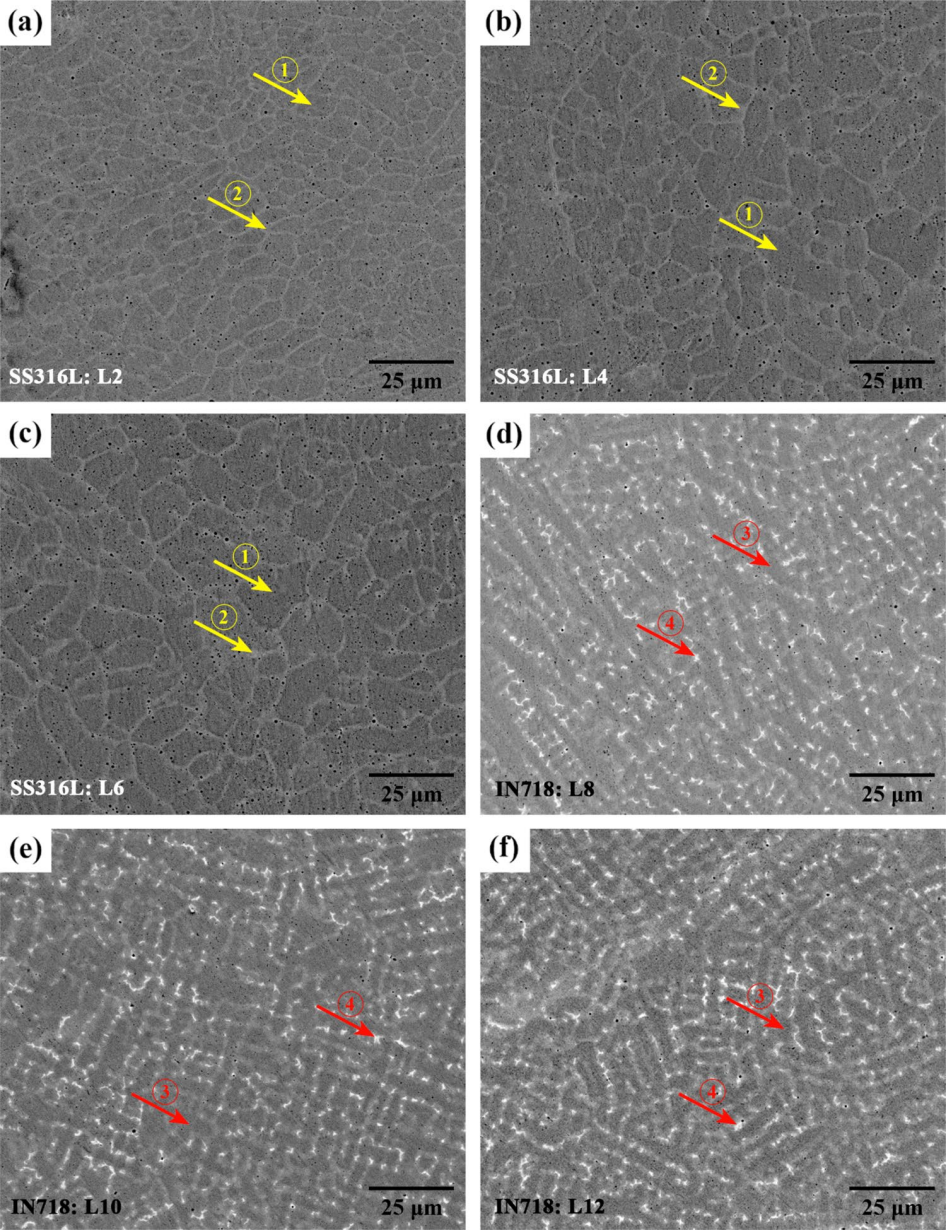
The cross-section microstructure of SS316L layers: (**a**) 2, (**b**) 4, (**c**) 6 and IN718 layers: (**d**) 8, (**e**) 10, and (**f**) 12. The numbered arrows show the different contrasts selected for EDS analysis and the possible phases.

Figure 11a shows the average cooling rate in the solidification range for each layer. As expected, as the height of structure increases, the cooling rate decreases in the build direction due to heat accumulation and extension of the melt pool. As well, the changes in the cellular/dendritic arm spacing $\lambda$ (µm) can be anticipated according to the average cooling rate in each layer using the following equation:

15 $$\lambda =b{(CR)}^{-n}$$

where $CR$ is the cooling rate (K/s), and $b$ and $n$ are the material constants with values of 80 and 0.33 for stainless steel and 39.8 and 0.3 for nickel-based superalloy, respectively^23^. As shown in Fig. 11b, consistent with the results obtained from the micrographs of corresponding layers (Fig. 12), the microstructure size (i.e. cellular/dendritic arm spacing, $\lambda$), in both SS316L and IN718 sections increased independently from one layer to another in the build direction, due to the reduction of cooling rate and as a result more time for growth. However, due to the occurrence of the eutectic reaction L → γ + Laves in the IN718 section (Fig. 12d–f), which will be further explained, a finer microstructure was formed in layers 8–14, despite following the above-mentioned trend. It is also noteworthy that in previous similar studies, a very fine microstructure in the range of 3–10 microns has been observed^23,24^.

**Figure 13:**
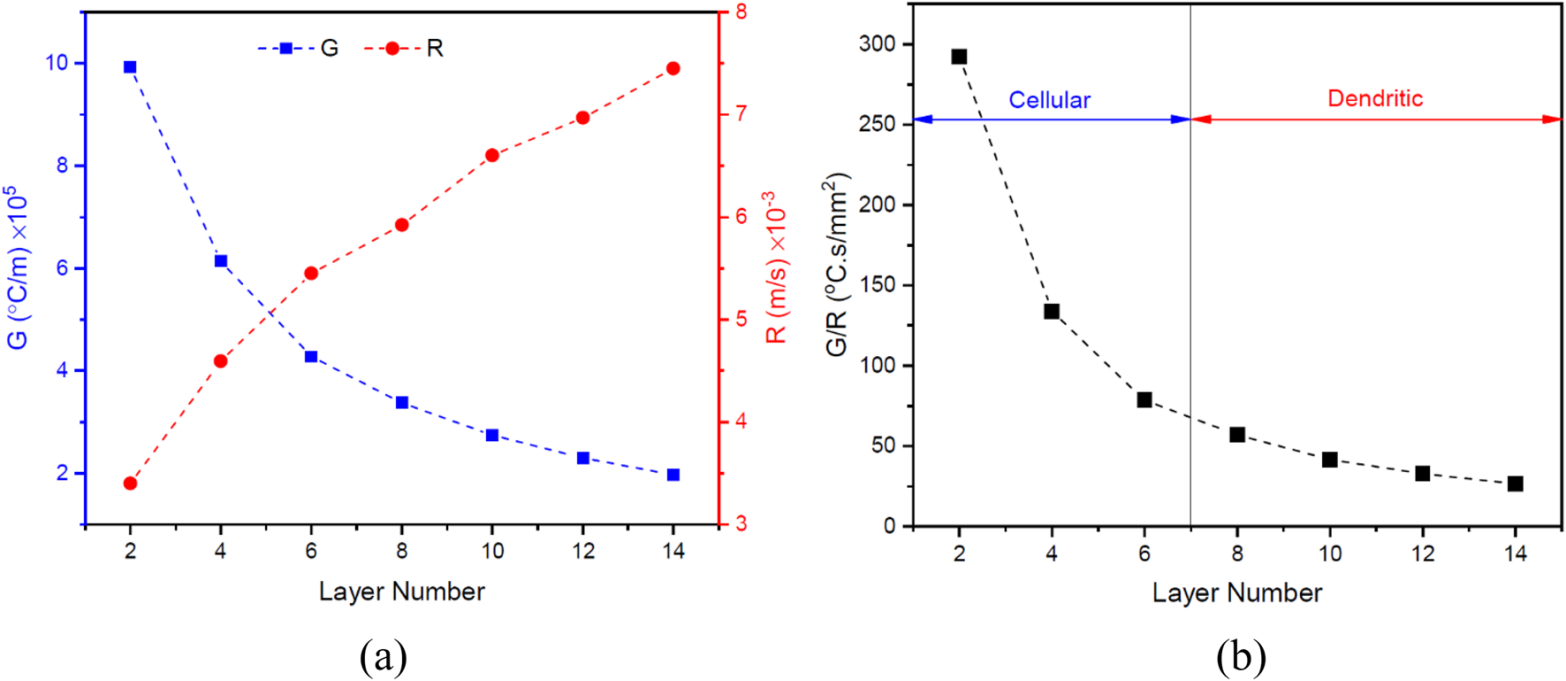
The variations of (**a**) temperature gradient ($G$) and solidification rate ($R$) and (**b**) undercooling ($G/R$) in the build direction.

**Figure 14:**
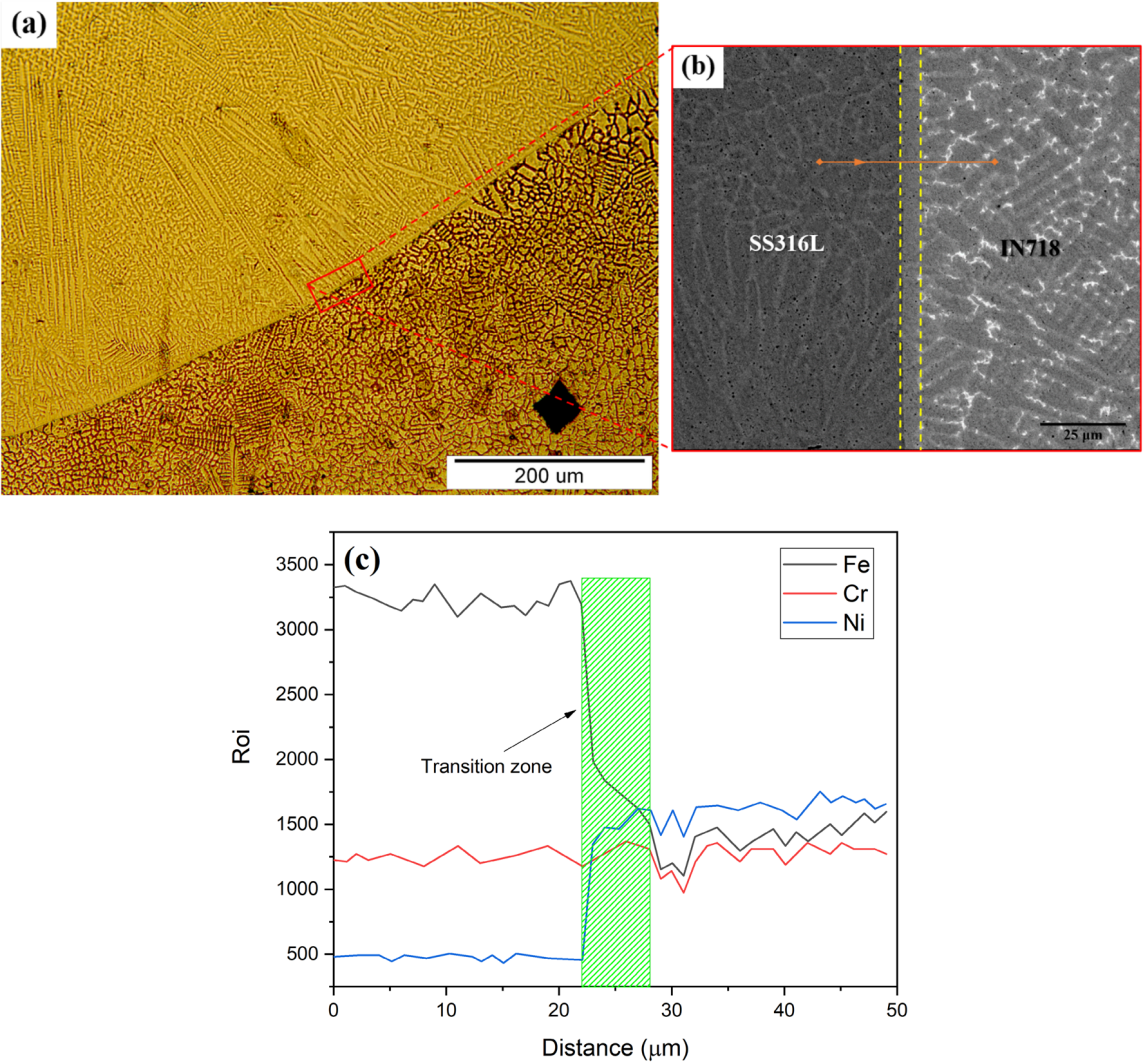
(**a**) Optical microstructure of the interface between layers 7 and 8. (**b**) SEM micrograph of the area specified in (**a**). The distance between the two dashed lines in (**b**) shows the range of planar solidification. (**c**) EDS line analysis along the specified path in (**b**) showing the transition zone at the interface.

On the other hand, by determining the temperature gradient ($G$) through the numerical model for each layer in the build direction and having the cooling rate in the relevant position, the advancing velocity of the solidification front or solidification rate ($R$) can also be calculated for each layer ($CR=G\cdot R$). Figure 13a shows that in the upper layers the temperature gradient decreases, and the solidification rate increases due to heat accumulation. Such changes along the structure height, by decreasing the $G/R$ ratio (Fig. 13b) can lead to an increase in undercooling and therefore more instability of the solidification front according to the following inequality^25^:

16 $$\frac{G}{R}\ge -\frac{{m}_{L}{C}_{s}^{*}\left(1-{k}_{0}\right)}{{k}_{0}{D}_{L}}$$

where ${m}_{L}$, ${C}_{s}^{*}$, ${k}_{0}$, and ${D}_{L}$ are the slope of the liquidus line, the solid composition at the interface, the distribution coefficient, and the diffusion coefficient in the melt, respectively. Although thermodynamic calculations can make a more accurate assessment, by changing the material to IN718 rich in various alloying elements, especially with low distribution coefficients (molybdenum and niobium), the ${C}_{s}^{*}$ and ${k}_{0}$ variables on the right side of inequality (16) increase and decrease respectively, both of which, in addition to the decrease in the $G/R$ ratio, cause further instability of the solidification front according to the inequality. As can be realized by comparing the micrographs presented in Fig. 10, the solidification morphology in layers 1–7 (SS316L section) is cellular and in layers 8–14 (IN718 section) is dendritic. However, at the almost defect-free interface of adjacent layers, as shown in Fig. 14a,b for the interface between layers 7 and 8, a planar solidification is observed in a short distance less than 10 µm. It is contrary to the general rule, which can be due to more local temperature gradient at the interface of two adjacent layers than the internal areas of each layer. It is also noteworthy here that the dilution effect between adjacent layers has led to a slight deviation from the primary multi-material design, and the formation of a transition zone and a kind of gradation at the interface of the two alloys, which can be observed by EDS line analysis in Fig. 14c and as reported in some previous studies^10,11^.

**Figure 15:**
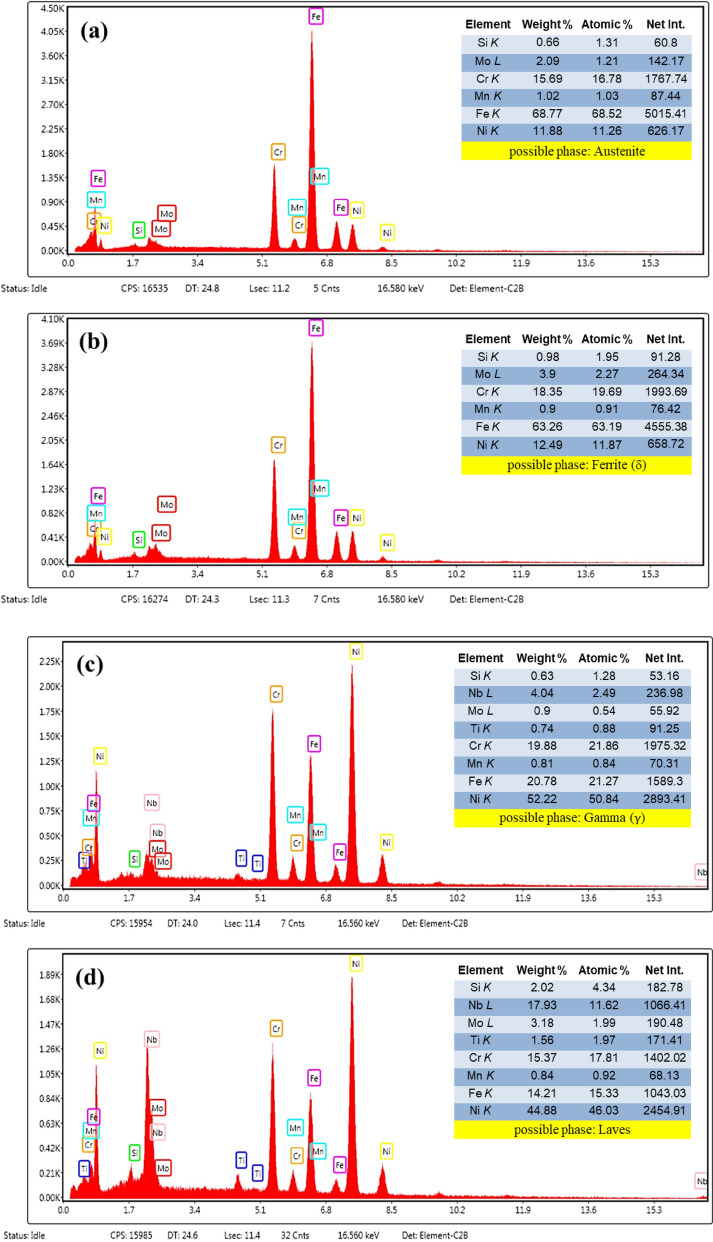
The results of chemical analysis and possible phases from the tip position the of arrows (**a**) 1 and (**b**) 2 in Fig. 12c and arrows (**c**) 3 and (**d**) 4 in Fig. 12f.

**Figure 16:**
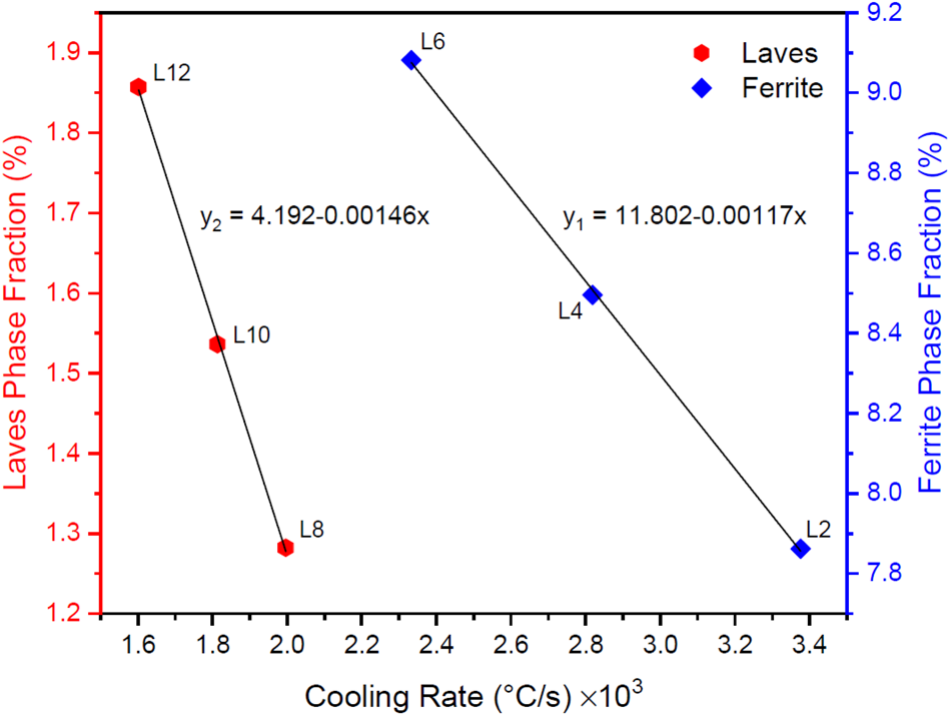
Variations in the fraction of ferrite and Laves phases with the cooling rate (during solidification) in different layers and the linear regression analysis of each.

Another characteristic feature of the microstructure of each layer is its chemical distribution and phase composition. Figure 15 shows a set of EDS analysis results from areas with different contrasts in the microstructures presented in Fig. 12 (numbered arrows). Considering the results and matching them with previous studies^26,27^, the multi-material microstructure in the SS316L section mostly consists of austenite phase and a small amount of the delta ferrite phase (δ) in the intercellular regions (Fig. 15a,b, respectively). Also, in the IN718 section, the microstructure consists of the gamma matrix phase (γ) and the intermetallic compound of Laves in the interdendritic regions (Fig. 15c,d, respectively). In fact, during non-equilibrium solidification in both sections, elements with lower distribution coefficients (Cr, Mo, and Si in SS316L and Nb, Mo, Si, and Ti in IN718) were segregated in the intercellular/dendritic regions, and by providing the necessary driving force, have led to the nucleation and growth of the mentioned secondary phases in the final stages of solidification. However, the size and distribution of the secondary phases are not uniform due to different cooling rates during solidification in different layers. As can be seen in Fig. 16, fraction of ferrite and Laves phases in layers 2–6 and 8–12, respectively, increased almost linearly with decreasing cooling rate. This is because by reducing the cooling rate, more time is provided for the diffusion of alloying elements and thus their microsegregation. It should be noted that the thermal cycles caused by the deposition of subsequent layers do not have a significant effect on the secondary phases resulting from solidification through solid-state diffusion, because the necessary temperature and time are not provided to modify or dissolve them^28^.

**Figure 17:**
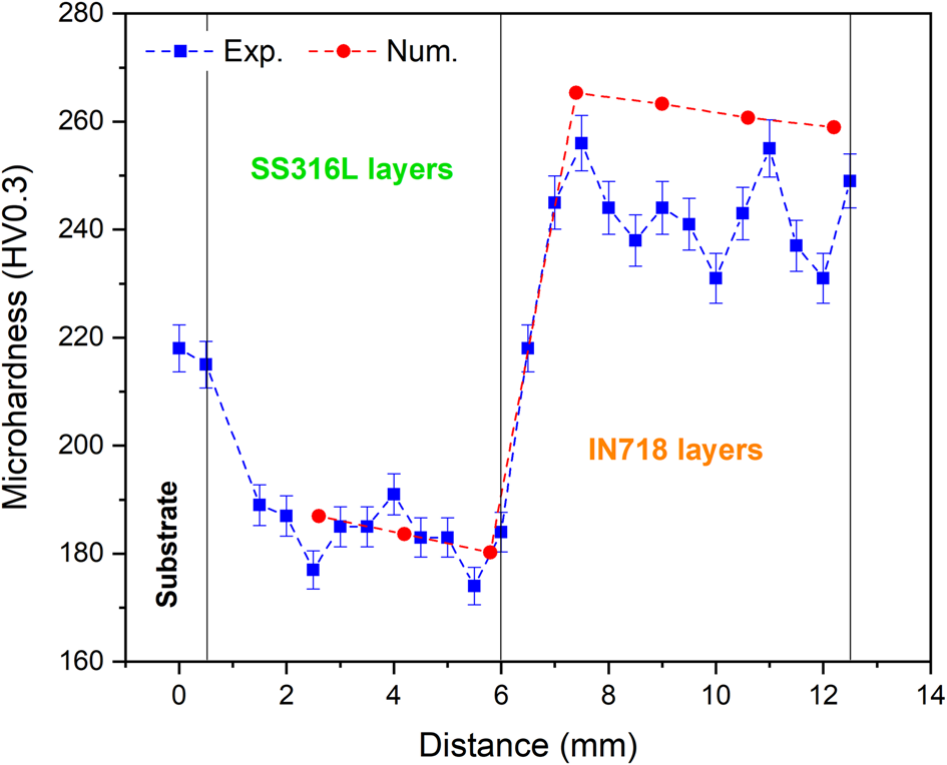
Microhardness variations along the cross-section of the multi-material structure.

It is possible to predict the hardness distribution along the structure using the thermal analysis results and the following relationships:

17 $${\sigma }_{y}={\sigma }_{0}+k({{d}_{g})}^{-0.5}$$

18 $$HV=3{\sigma }_{y}{(0.1)}^{m-2}$$

where in Eq. (17), known as the Hall–Patch relationship, ${\sigma }_{y}$, ${\sigma }_{0}$, $k$, and ${d}_{g}$ are the yield stress (MPa), friction stress (MPa), locking parameter (Mpa µm^−1/2^), and grain size (µm), respectively. There is no doubt that the grain size is different from the cell/dendrite size of the solidification structure, but for the additive manufactured samples, good compatibility has been observed in the Hall-Patch relationship when grain size (${d}_{g}$) is replaced with cell/dendrite size (*λ*)^24^. Therefore, the cell/dendrite size was used instead of the grain size in this prediction. The constants ${\sigma }_{0}$ and $k$ were also considered to be 194 MPa and 695 Mpa µm^-1/2^ for SS316L^29^ and 325 MPa and 750 Mpa µm^−1/2^ for IN718^30^, respectively. In Eq. (18), $HV$ and $m$ are Vickers hardness (kgf/mm^2^) and Mayer’s index, respectively, and $m$ was considered to be 2.25 for both alloys^24^. Figure 17 illustrates the microhardness distribution along the cross-section of the multi-material structure by experimental measurement and numerical calculation. As shown, there is a fair correlation between the results obtained from the two methods, though the influence of other strengthening mechanisms (solid solution and secondary phase strengthening) which are not directly included as independent terms in Eq. (17), can be considered as the reason for the difference between the numerical and experimental results, especially in the IN718 section. A further explanation that, in the SS316L section, despite the expected hardness drop from layers 1–7 due to the increase in microstructure size, it is almost uniform. This can be due to equal competition between the two mechanisms of hardness reduction (microstructure coarsening) and hardness increase (ferrite phase reinforcement by decreasing the cooling rate) in this section with increasing the structure height. However, with the transition to the IN718 section, the hardness increases significantly due to higher yield strength of the matrix phase and the presence of the Laves intermetallic phase, and then, it decreases slightly with the coarsening of the microstructure in layers 8–14.

## Conclusion

In this research, with the aim of comprehensive understanding and the possibility of predicting different aspects of the materials paradigm in additive manufacturing of multi-materials, the printability and microstructure evolution of SS316L-IN718 multi-material fabricated by the directed energy deposition method were studied through numerical modeling and experimental analysis. The main results are as follows:

1. Printability analysis of the multi-material showed that, in general, distortion and composition change defects are more pronounced with increasing the number of layers due to heat accumulation. In contrast, lack of fusion is more likely to happen in the initial layers due to more efficient heat loss through the substrate.
2. Due to the difference in the thermophysical properties of the base materials, the IN718 section with a maximum thermal strain of 3.95 × 10^−3^ in the last layer is more sensitive to distortion, and on the other side, the SS316L section with vaporization of more than 0.9% of Mn element in the sixth layer and also the lack of fusion index close to 1 in the initial layers is more susceptible to composition change and lack of fusion.
3. Although with the progress of the deposition process, a coarser solidification structure is formed due to the reduction of cooling rate, it was shown experimentally and numerically that the occurrence of the eutectic reaction L → γ + Laves in the IN718 section causes this rule to be discriminated by a finer microstructure in layers 8–14, despite following the general trend.
4. Calculation of the ratio of temperature gradient ($G$) and solidification rate ($R$) in the build direction and matching it with the relevant microstructures showed that the cellular solidification of SS316L section can be separated from the dendritic solidification of IN718 section by decreasing the $G/R$ ratio (increasing undercooling) to approximately 55 K s/mm^2^, besides the influence of increasing the concentration of alloying elements with low distribution coefficients. However, a very narrow planar solidification mode is also visible at the interface of adjacent layers due to the higher local gradient temperature.
5. The fraction of secondary phases (delta ferrite in SS316L and Laves in IN718) resulting from non-equilibrium solidification in the intercellular/dendritic regions increases almost linearly (with different slopes) due to the reduction of cooling rate in the build direction of the multi-material.
6. The prediction and measurement of hardness in the build direction similarly showed the highest hardness in the IN718 section (~ 260 HV0.3) due to higher yield strength of the matrix and the presence of the Laves intermetallic phase. Nevertheless, the hardness variations in each multi-material section with increasing number of layers, due to microstructure coarsening (hardness reduction factor) along with secondary phase reinforcement (hardness increase factor), has been mild and somewhat decreasing.

## Data Availability

All data generated or analyzed during this study are included in this published article.

## References

[1] DebRoy T (2018). Additive manufacturing of metallic components–process, structure and properties. Prog. Mater. Sci..

[2] Bandyopadhyay A, Heer B (2018). Additive manufacturing of multi-material structures. Mater. Sci. Eng. R. Rep..

[3] DebRoy T (2019). Scientific, technological and economic issues in metal printing and their solutions. Nat. Mater..

[4] Ghanavati R, Naffakh-Moosavy H (2021). Additive manufacturing of functionally graded metallic materials: A review of experimental and numerical studies. J. Market. Res..

[5] Feenstra DR (2021). Critical review of the state of the art in multi-material fabrication via directed energy deposition. Curr. Opin. Solid State Mater. Sci..

[6] Rodriguez J, Hoefer K, Haelsig A, Mayr P (2019). Functionally graded SS 316L to Ni-based structures produced by 3D plasma metal deposition. Metals..

[7] Lin X, Yue TM, Yang HO, Huang WD (2005). Laser rapid forming of SS316L/Rene88DT graded material. Mater. Sci. Eng. A.

[8] Lin X, Yue TM (2005). Phase formation and microstructure evolution in laser rapid forming of graded SS316L/Rene88DT alloy. Mater. Sci. Eng. A.

[9] Shah K (2014). Parametric study of development of Inconel-steel functionally graded materials by laser direct metal deposition. Mater. Des..

[10] Savitha U (2015). Chemical analysis, structure and mechanical properties of discrete and compositionally graded SS316–IN625 dual materials. Mater. Sci. Eng. A.

[11] Zhang X, Chen Y, Liou F (2019). Fabrication of SS316L-IN625 functionally graded materials by powder-fed directed energy deposition. Sci. Technol. Weld. Joining.

[12] Carroll BE (2016). Functionally graded material of 304L stainless steel and inconel 625 fabricated by directed energy deposition: Characterization and thermodynamic modeling. Acta Mater..

[13] Su Y, Chen B, Tan C, Song X, Feng J (2020). Influence of composition gradient variation on the microstructure and mechanical properties of 316 L/Inconel718 functionally graded material fabricated by laser additive manufacturing. J. Mater. Process. Technol..

[14] Kim SH (2021). Selective compositional range exclusion via directed energy deposition to produce a defect-free Inconel 718/SS 316L functionally graded material. Addit. Manuf..

[15] Manvatkar VD, Gokhale AA, Reddy GJ, Savitha U, De A (2015). Investigation on laser engineered net shaping of multilayered structures in H13 tool steel. J. Laser Appl..

[16] Goldak JA, Akhlaghi M (2006). Computational Welding Mechanics.

[17] Davis JR (1994). Stainless Steels.

[18] Davis JR (2000). Nickel, Cobalt, and Their Alloys.

[19] De A, DebRoy T (2004). A smart model to estimate effective thermal conductivity and viscosity in the weld pool. J. Appl. Phys..

[20] Mukherjee T, Zuback JS, De A, DebRoy T (2016). Printability of alloys for additive manufacturing. Sci. Rep..

[21] Lippold JC (2014). Welding Metallurgy and Weldability.

[22] Mukherjee T, DebRoy T (2019). Printability of 316 stainless steel. Sci. Technol. Weld. Joining.

[23] Zheng B, Zhou Y, Smugeresky JE, Schoenung JM, Lavernia EJ (2008). Thermal behavior and microstructure evolution during laser deposition with laser-engineered net shaping: Part II. Experimental investigation and discussion. Metall. Mater. Trans. A..

[24] Manvatkar V, De A, DebRoy T (2014). Heat transfer and material flow during laser assisted multi-layer additive manufacturing. J. Appl. Phys..

[25] Flemings MC (1974). Solidification. Metall. Mater. Trans. B..

[26] Zhang CH (2017). Multi-layer functional graded stainless steel fabricated by laser melting deposition. Vacuum.

[27] Ghanavati R, Naffakh-Moosavy H, Moradi M (2021). Additive manufacturing of thin-walled SS316L-IN718 functionally graded materials by direct laser metal deposition. J. Market. Res..

[28] Kumara C, Balachandramurthi AR, Goel S, Hanning F, Moverare J (2020). Toward a better understanding of phase transformations in additive manufacturing of Alloy 718. Materialia..

[29] Wang Z, Palmer TA, Beese AM (2016). Effect of processing parameters on microstructure and tensile properties of austenitic stainless steel 304L made by directed energy deposition additive manufacturing. Acta Mater..

[30] Gallmeyer TG (2020). Knowledge of process-structure-property relationships to engineer better heat treatments for laser powder bed fusion additive manufactured Inconel 718. Addit. Manuf..

